# Factors Associated With Loneliness During the COVID-19 Pandemic Among Older Adults With Chronic Conditions

**DOI:** 10.1093/geroni/igaa057.3462

**Published:** 2020-12-16

**Authors:** Courtney Polenick, Emily Perbix, Shreya Salwi, Donovan Maust, Kira Birditt, Jessica Brooks

**Affiliations:** 1 University of Michigan, Ann Arbor, Michigan, United States; 2 Columbia University, New York, New York, United States

## Abstract

Social distancing related to the COVID-19 pandemic may heighten loneliness among older adults, especially those with chronic conditions that increase risk for severe illness from the coronavirus. Little is known, however, about potential risk and protective factors linked to loneliness during the pandemic. In the present study, we examined factors associated with loneliness in a U.S. sample of adults aged 50 and older with at least one chronic condition. Participants included 701 adults aged 50 to 94 (M = 64.57 years, SD = 8.84) who were recruited over 8 consecutive weeks between May 14 and July 9, 2020 to complete an anonymous online survey. We estimated a series of multiple linear regressions to determine how sociodemographic characteristics, health characteristics, stress related to COVID-19, and social resources were independently associated with loneliness during the pandemic. Two-thirds of participants reported moderate to severe loneliness. The fully adjusted regression model showed that being a person of color, having a spouse or cohabiting partner, and reporting more emotional support were linked to lower levels of loneliness. Higher anxiety symptoms, more worry about being infected with COVID-19, and greater perceived financial strain because of COVID-19 were associated with higher levels of loneliness. These findings pinpoint potential targets for interventions to improve and maintain the well-being of a particularly vulnerable subgroup of older adults during the COVID-19 pandemic and in future public health crises.

